# Assessing Health Care Professionals' Mindset in Adopting Telemedicine Post COVID-19: Pilot Questionnaire Study

**DOI:** 10.2196/44806

**Published:** 2023-06-02

**Authors:** Rozhin Naghdi, Gianhu Nguyen, Cecile Maria Vazquez, Christian Mark Antonio, Carlos Cabrera, Austin Chandra, Jay Chok

**Affiliations:** 1 Keck Graduate Institute Claremont, CA United States

**Keywords:** digital health, patient perspective, creative destruction of medicine, pilot study, patient-centered health, willingness, delivery model, questionnaire, telehealth, accessibility, implementation

## Abstract

**Background:**

Amidst the COVID-19 pandemic, the traditional health care model has evolved toward a more patient-centric model. In relation to this trend, digital health services have seen an acceleration, which may have significant implications for the health care model. Due to the impact of COVID-19 on health care facilities, it is important to explore health professionals’ willingness to adopt a patient-centric digital health delivery model for medicine and health care.

**Objective:**

The aim of this study was to pilot a survey that assesses the impact and implementation of telehealth in view of health care providers prior to and post COVID-19.

**Methods:**

A total of 26 volunteer health care professionals participated in the pilot study, of which 19/26 (73%) completed the general demographics portion. Among these respondents, 9/26 (35%) completed the entirety of the survey. The questionnaire included questions relating to general demographics, accessibility and benefits, usability, and engagements with telemedicine. Participants were randomly assigned to 1 of the 4 questionnaire designs (A-D) based on their expertise in telehealth. Of the 9 total participants who completed their randomly assigned questionnaire, 1 (11%) was randomly assigned to A, 3 (33%) were randomly assigned to B, 2 (22%) were randomly assigned to C, and 3 (33%) were randomly assigned to D.

**Results:**

Responses and data from the study questionnaire were collected from Qualtrics. Microsoft Excel was used for data organization. Due to limited responses and data, no advanced statistical software was implemented. From the 9 participants who completed the entirety of the survey, responses from those with telehealth experience (n=4) showed that telehealth was preferred for follow-ups, lab results, and consultations, and that with telehealth, there was greater flexibility with appointment times and a decrease in the number of patients seen. Among the 4 health care providers with telehealth experience, all of them believed it improved accessibility and reduced physical barriers; health care professionals believed telehealth reduced translational barriers with patients. Among health care professionals without telehealth experience (n=5), 60% (3/5) reported a decrease in appointments for in-office visits post COVID-19 and strongly agreed or agreed that telehealth could influence the quality of care for patients. All 5 participants also reported no general concerns about telehealth prior to the pandemic and agreed that it would provide accessibility for patients.

**Conclusions:**

Preliminary findings of our pilot study showed initial support of a dynamical shift within the health care model due to the rise in the use of telehealth services between health care providers and patients but no statistically significant results. Further research and investigation with a larger sample size is warranted to better understand the mindset of health care professionals in adopting telemedicine post COVID-19.

## Introduction

### Background

In recent years, the Food and Drug Administration has classified digital health broadly to include telehealth, digital medicine, mobile health, health information technology, and other forms of personalized medicine that use different aspects of digital technologies to deliver quality health care services [1]. The growth of digital health technologies has been theorized to arise from the growth in technological advancements, and the next step seems to allude to how health care systems plan to use digital health [2-6]. For example, the COVID-19 pandemic presented health care systems with many challenges in delivering safe and effective patient care while reducing the spread of transmission [7]. Digital health can overcome these challenges but requires providers to shift practicing models from direct contact toward telemedicine [7,8]. Increasingly, there is also a need to incorporate patients’ perspectives into digital health design [9].

Data from recent studies suggest that the COVID-19 pandemic has largely accelerated the need to rethink the implications of digital health in existing health care systems [3-5,10]. For example, Bordoloi et al [11] documented the growing trend of patient engagement and satisfaction increasing as provider interaction becomes increasingly simpler and more efficient. As a result, patient populations are asking their providers more extensive medical questions, defending their rights and willingness as a patient, and desiring a more active role in their treatment plan [2]. Narayanan [12] likewise reported that patients with chronic conditions value digital health modalities for routine health checks, treatment communication checks, and wellness checks with their health care professionals. This type of thinking has the potential to lead to a breakdown of the medical hierarchy within the provider-patient relationship in favor of more personalized medicine [2,8].

In addition, the use of digital health can allow for a shift toward personalized medicine by reducing inefficiencies, improving access, reducing costs, and increasing the quality of patient care [1,2]. An example of this can be seen in the work of Hirko et al [13], who explored the potential benefits of digital health and telehealth programs in addressing rural health disparities during the COVID-19 pandemic. Their work shows that nearly 14,000 digital health visits were accomplished within 6 weeks, which offered safety measures for both patients and practicing physicians. Hirko et al [13] also commented on the need to develop a sustainable infrastructure for digital health in these areas to overcome bandwidth challenges among other issues. Therefore, it would be beneficial to analyze how the pandemic has accelerated this emergent health care model, including the potential for improved patient care quality associated with digital health. Doing so could provide great insights about the future of health care.

### Study Goal

This study is designed as a pilot data research study on the impact of telemedicine in Southern California. To that end, respondents serve to give a valuable perspective on the implications of digital health in view of the COVID-19 pandemic across all medical specialties.

## Methods

### Ethics Approval

This pilot research protocol was submitted to the institutional review board (IRB) at Claremont Graduate University (CGU #4188) and approved by a representative of the IRB on April 6, 2022.

### Participants

To recruit participants for our pilot study, we used the Health Resources and Service Administration website and created a contact list. We reached out to 499 health centers and clinics within a 50-mile range of the city of Claremont, California. Following the recruitment protocol, the invitation was extended nonverbally via emails and orally through web-based and in-person communications.

### Questionnaire Design

The questionnaire was formulated following a focus group with 2 experts in the telemedicine field. To ensure the questionnaire accurately addresses research questions, the experts provided detailed information about current integration of telemedicine, costs, and key obstacles prior to and post the COVID-19 pandemic. This pilot study tests the clarity and comprehension of the questions as well as the consent form. Responses were collected over a 3-month period.

**Figure 1:**
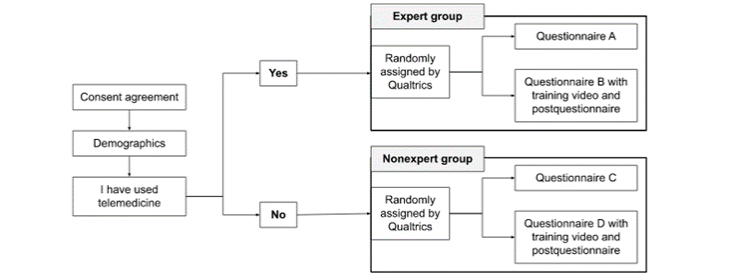
Questionnaire assignment breakdown.

The questionnaire consisted of 4 sections related to general demographics, accessibility and benefits, usability, and engagements with telemedicine within 2 groups—experts and nonexperts (Figure 1). The expert group was further divided into 2 groups—questionnaire A without the training video and questionnaire B with the training video and a postquestionnaire. Questionnaire A consisted of 25 questions and questionnaire B with the postquestionnaire consisted of a total of 56 questions. The expert group postquestionnaire assessed the view of current applications of telehealth and future considerations post the COVID-19 pandemic.

Similarly, the nonexpert group was further divided into 2 groups—questionnaire C without the training video and questionnaire D with the training video and a postquestionnaire. Questionnaire C consisted of 15 questions and questionnaire D with the postquestionnaire consisted of a total of 31 questions. Participants viewed the training video through the video incorporation feature of Qualtrics within the questionnaire. Those randomly assigned to groups with the training video, in addition to completing the same initial questionnaire as their respective groups, also answered a postquestionnaire. The nonexpert group postquestionnaire assessed the health care provider’s view in adopting telehealth in the future and the comprehension of the benefits and risks.

The training video explained applications of telehealth, such as removing language and cost barriers, and its risks and benefits to both the patient and the health care provider. It provided a broad context of the integration of telemedicine in patient-centric health care models. The training video was obtained from Facebook; however, it is no longer available for viewing. We will provide the training video upon request.

The issues observed by participants in this pilot study questionnaire were (1) the length of the questionnaire and the training video (too long) and (2) difficulty in the use of a web-based survey platform (Qualtrics).

### Procedures

The questionnaire was sent to the participants after confirming their interest and intent to participate in the study. They were able to complete the web-based questionnaire on Qualtrics, a cloud-based survey software, at the time and place of their convenience. Participants with telemedicine experience were randomly assigned by Qualtrics to either questionnaire A, with no training video, or questionnaire B, with a training video and postquestionnaire. Participants with no telemedicine experience were randomly assigned by Qualtrics to either questionnaire C, with no training video, or questionnaire D, with a training video and postquestionnaire.

### Data Collection and Measures

Responses and data from the study questionnaire were collected from Qualtrics. Microsoft Excel was used for data organization. Due to limited data, advanced statistical software was not required, and no substantial analyses were made. Through the health care professionals’ experience and feedback, the questionnaire contained primary measures of general demographics, accessibility and benefits, usability and barriers, and engagement with telemedicine.

## Results

A total of 26 health care professionals participated in the study across all questionnaire arms with 19/26 (73%) completion rate in the general demographics portion. Among these respondents, 9/26 (35%) completed the entirety of the questionnaire after being randomly assigned by Qualtrics to questionnaires A, B, C, or D, with an average completion time of 12.8 minutes, while 10/26 (38%) only answered general demographic questions and discontinued after an average time of 1.1 minutes. Of the 9 total participants who completed their randomly assigned questionnaires, 1 (11%) was randomly assigned to questionnaire A, 3 (33%) were randomly assigned to questionnaire B, 2 (22%) were randomly assigned to questionnaire C, and 3 (33%) were randomly assigned to questionnaire D.

Of the participants who completed the general demographics section, 10/19 (53%) were female, 8/19 (42%) were male, and 1/19 (5%) did not report sex (Table 1). The age range of health care professionals were as follows: 2/19 (11%) between 18 and 24 years, 10/19 (53%) between 25 and 40 years, 5/19 (26%) between 41 and 56 years, 3/19 (16%) between 57 and 75 years, and 0/19 (0%) between 76 and 93 years. Health care professions were classified in the study under the following types: medical doctor (5/19, 26%), physician assistant (2/19, 11%), nurse practitioner (2/19, 11%), registered nurse (5/19, 26%), dentist (1/19, 5%), and other health care professions (5/19, 26%) specified later; 8/19 (42%) specialized in primary care, 1/19 (5%) specialized in pediatrics, 1/19 (5%) specialized in psychiatry, 3/19 (16%) specialized in internal medicine, 0/19 (0%) specialized in obstetrics and gynecology, 1/19 (5%) specialized in surgery, and 6/19 (32%) specialized in other departments not listed. Of the 5/19 (26%) other professions, 2/5 were medical assistants, 1/5 was a phlebotomist, 1/5 was a medical billing specialist, and 1/5 was a telehealth clinic manager; of the 5/19 (26%) other specialists, 2/5 specialized in medical research, 1/5 specialized in gastroenterology, 1/5 specialized in medical insurance, and 1/5 did not specify.

A total of 9/19 (47%) had experience using telehealth and were randomly assigned to either questionnaire A or B, while 10/19 (53%) did not have any prior experience using telehealth and were randomly assigned to either questionnaire C or D. Of those with telehealth experience, 3/9 (33%) worked in a federal clinic or hospital, 1/9 (11%) worked in a nonprofit hospital or clinic, 3/9 (33%) worked in a private hospital or clinic, and 2/9 (22%) worked in a university hospital or clinic. Of those without telehealth experience, 1/10 (10%) worked in a federal hospital or clinic, 1/10 (10%) worked in a free clinic, 4/10 (40%) worked in a private hospital or clinic, 3/10 (30%) worked in private practice, and 1/10 (10%) worked in a university hospital or clinic.

**Table 1:** Demographic characteristics of the overall pilot study sample.

Demographics		Overall (n=19), n (%)		Participants who completed the questionnaire (n=9), n (%)
**Sex**				
	Male	8 (42)	4 (44)	
	Female	10 (53)	5 (56)	
**Age (years)**				
	18-24	2 (11)	2 (22)	
	25-40	10 (53)	5 (56)	
	41-56	5 (26)	0 (0)	
	57-75	3 (16)	2 (22)	
	76-93	0 (0)	0 (0)	
**Profession**				
	Medical doctor	5 (26)	3 (33)	
	Physician assistant	2 (11)	0 (0)	
	Nurse practitioner	2 (11)	1 (11)	
	Registered nurse	5 (26)	1 (11)	
	Dentist	1 (5)	0 (0)	
	Other	5 (26)	4 (44)	
**Specialty**				
	Primary care	8 (42)	2 (22)	
	Pediatrics	1 (5)	0 (0)	
	Psychiatrics	1 (5)	1 (11)	
	Internal medicine	3 (16)	2 (22)	
	Obstetrics and gynecology	0 (0)	0 (0)	
	Surgeon	1 (5)	1 (11)	
	Other	6 (32)	3 (33)	
**Professional experience (years)**				
	0-5	6 (32)	5 (56)	
	6-10	5 (26)	1 (11)	
	10-15	1 (5)	0 (0)	
	15-20	4 (21)	2 (22)	
	≥20	2 (11)	1 (11)	
**Health center**				
	Federal clinic or hospital	4 (21)	1 (11)	
	Free clinic	1 (5)	1 (11)	
	Nonprofit hospital or clinic	1 (5)	0 (0)	
	Private hospital or clinic	7 (37)	4 (44)	
	Private practice	3 (16)	1 (11)	
	University-affiliated clinic or hospital	3 (16)	2 (22)	
**Patient annual income bracket (US $)**				
	0-24,000	8 (42)	3 (33)	
	25,000-77,000	6 (32)	2 (22)	
	78,000-190,000	1 (5)	0 (0)	

## Discussion

### Principal Findings

The preliminary results of our pilot study show initial support of a dynamical shift within the health care model due to the rise in the use of telehealth services between health care providers and patients; however, a full study is needed to further support this view. This mirrors ideas introduced by Meskó et al [2], where patients play a much more active role while interacting with their health care provider as compared to older, traditional models [2,8]. For example, among those with telehealth experience, 3/4 (75%) health care professionals strongly agree that telehealth provides greater flexibility with appointment times, and 3/4 (75%) report a decrease in the number of patients seen through telehealth as compared to in-office visits; 4/4 (100%) prefer follow-ups, lab results, and consultations to happen through telehealth. These results demonstrate the potential for an increase in overall flexibility occurring between patients and their providers. This could be indicative of a shift toward a more personalized health care model that places more emphasis on the patients’ time [2,8]. The further use of telehealth services in health care could further influence this shift to a tailored health care for the patient. Additionally, on the measure of barriers, 4/4 (100%) strongly agree or agree that translational and accessibility barriers are reduced through telehealth, while the same quality of care is maintained. As proposed by Meskó et al [2], the traditional model would focus on a distinct hierarchical relationship between the provider and the patient, where the provider—typically the physician—would hold authority and power and is the main decision maker for the patient’s health care plan. Thus, the patient would often adhere to conditions and have to overcome barriers that maintained this hierarchy. Patients would be expected to go to a point-of-care site comfortable for the physician, like their office, for appointments, visits, and follow-ups [2]. However, the preliminary results show that providers using telehealth believe it to improve accessibility and reduce the physical barriers that patients would normally have to overcome. The results also show that health care professionals believe telehealth reduces translational barriers with patients, which might infer a language component as a possible testing variable for future research. Interestingly, among health care professionals without telehealth experience, 3/5 (60%) participants reported a decrease in appointments for in-office visits post COVID-19. This supports how telehealth implementation allows patients to overcome certain barriers to accessibility of patient care, and it further shows a possible correlation with findings by Meskó et al [2]. They also reported no general concerns about telehealth prior to COVID-19. In total, 5/5 (100%) strongly agree or agree with the accessibility that telehealth would provide for patients, and 3/5 (60%) strongly agree or agree that telehealth could influence the quality of care for patients. Therefore, the traditional, hierarchical model could be on the cusp of being replaced with a collaborative model between provider and patient without weakening the quality of care for the patient. Within this collaborative model, the patient can make active decisions for their care with physicians acting more like “guides,” as Meskó et al [2] would describe it. The shift in the health care model would have further implications for patient participation, expectations of providers, and other conditions surrounding the provider-patient relationship that could alter future patient care delivery.

A possible contrasting viewpoint was also found in the data. Of the participants who watched the training video, 3/3 (100%) were neutral on the cost-benefits of telehealth for both patients and providers, quality of care, and the future of telehealth in medicine. Therefore, even though all participants believed telehealth would improve accessibility, their views on telehealth improving patient care did not always align. Some physicians and providers may hold strong beliefs that the traditional health care model is optimal for providing quality patient care. This could warrant a follow-up study to test what types of differences correlate to whether health care professionals would favor either health care model. This study could measure variables in age, area of health care practice, patient demographics, or other factors.

### Limitations

Although this pilot study provides preliminary insights on telemedicine integration across different health specialties and disciplines, there are limitations to this study, particularly relating to the recruitment of human subjects as well as questionnaire design and completion rate. The limitations of this study include (1) receiving permission from department directors for health care providers to participate, (2) lack of commitment and communication from medical facilities and personnel, (3) lack of motivation to participate due to no incentives, and (4) outdated contact and facility information on the Health Resources and Service Administration website.

#### Recruitment

The pilot study recruitment proceeded with email invitations and individual emails to specific medical facilities—a mailing list of 499. The team also extended the invitation to local academic institutions, including Western University and the University of La Verne.

The first step of recruitment was initiated with emails and followed with a phone call within a week after the email invitation. Once interest in participating was confirmed, Zoom meetings were scheduled to provide an overview of the study and instructions. However, the overall lack of respondents and direct contact with potential study participants made it difficult to obtain data. Additionally, the participation of professionals from some health care institutions required approval from the board of directors. Participants’ desire for a more high-touch approach suggests the need to include individualized guidance for higher rate of responses and completion.

#### Questionnaire Design and Completion Rate

Our designed questionnaire measures multiple variables, and therefore, the length of each questionnaire, including the general demographic section, varies from 38 to 69 questions, which can take 15 to 30 minutes to complete. However, the time taken to complete the questionnaire varied among the individuals who finished the questionnaire between 1.2 and 19.5 minutes. This suggests that modifications should be made to the questionnaire in a manner that would reflect the intended completion time frame across participants. As a mitigative measure, creating multiple shorter questionnaires testing 1 or 2 variables and randomly assigning participants to each one may address this challenge.

Another limitation is that all questions were made optional for participants to answer. Participants were not required to answer all questions to move on to the next question, resulting in missing responses. We allowed respondents to skip questions that they did not feel comfortable answering. This was done initially to offer participants more freedom surrounding their answers. However, this greatly impacted the overall completion rate of the questionnaire, going from 73% (19/26) to 35% (9/26).

### Future Research

Our initial pilot study suggests that cold outreaching may not be the best approach for this population. Most health care professionals were not responsive to our outreach modality. Through the challenges of recruiting participants for this pilot study, the team reflected and now believes offering continuing medical education (CME) credits is a potential approach to increasing interest from professionals in the health care field. This suggests the need for expansion and improvement in the methodology. CME credits would mitigate the issues related to human subject recruitment and would be a valuable incentive for quality assurance improvement. To execute the survey more successfully with higher rates of completion in this demographic, we suggest breaking metrics and variables into multiple surveys rather than one long survey. In a CME setup, the team members will be available for assistance and to answer any questions from participants.

Along these lines, it is important to create a CME-appropriate training, including concept videos, that meet the intended objective of providing an optimal avenue to analyze health care professionals’ interpretations. Assessment procedures should be implemented to explore how the training video aids the health care professionals to understand the impact of telehealth services on quality patient care. CME-appropriate training can have 2 arms that facilitate obtaining the patient perspective for the trainee. In the first arm, trainees learn directly from the instructor about various aspects of telehealth, such as patient satisfaction, technology challenges, as well as cultural and socioeconomic perception of telehealth. In the second arm, participants are trained with a qualitative focus group methodology with the training team helping to simulate the patient perspective. It is important that the trainee is briefed beforehand with no deception. Otherwise, the IRB proposal could be problematic.

Finally, the choice of a conference venue for providing the CME needs to be thought through. Some health care conferences are more regional. It is important to consider whether a national conference can provide a larger sample size of health care professionals due to a broader geographical outreach.

### Conclusions

This pilot study serves to assess the integration of telemedicine and digital health by health care providers. Both experienced and nonexperienced participants favored telemedicine for the improved accessibility and reduced barriers post COVID-19 for specified appointment types; however, nonexperienced providers remained neutral in incorporating telemedicine technology into their practice and further disagreed on the quality of care. Further research with a larger sample size is needed to better understand this view.

## Abbreviations

CME: continuing medical education
IRB: institutional review board

## Data Availability

Anonymous responses from the study questionnaire along with the training videos used during this pilot study are available from the corresponding author upon request.
